# The Association of Assisted Reproductive Technology with Placental and Umbilical Abnormalities

**DOI:** 10.3390/jpm15050176

**Published:** 2025-04-27

**Authors:** Antonios Siargkas, Ioannis Tsakiridis, Sonia Giouleka, Petya Chaveeva, Maria Mar Gil, Walter Plasencia, Catalina De Paco Matallana, Efstratios M. Kolibianakis, Themistoklis Dagklis

**Affiliations:** 1 3rd Department of Obstetrics and Gynecology, School of Medicine, Faculty of Health Sciences, Aristotle University of Thessaloniki, Agiou Dimitriou, 54124 Thessaloniki, Greecegkiouleka@auth.gr (S.G.); 2 Department of Obstetrics and Gynecology, Faculty of Medicine, Medical University of Pleven, 5800 Pleven, Bulgaria; chaveevapetya@gmail.com; 3 Fetal Medicine Unit, Dr. Shterev Hospital, 1330 Sofia, Bulgaria; 4 School of Medicine, Universidad Francisco de Vitoria, 28223 Madrid, Spain; 5 Department of Obstetrics and Gynecology, Hospital Universitario de Torrejón, 28850 Madrid, Spain; 6 Fetal Medicine Unit, Department of Obstetrics and Gynecology, Complejo Hospitalario Universitario de Canarias, 38320 Santa Cruz de Tenerife, Spain; 7 Institute for Biomedical Research of Murcia, IMIB—Arrixaca, El Palmar, Faculty of Medicine, Universidad de Murcia, 30120 Murcia, Spain; 8 Maternal Fetal Medicine Unit, Department Obstetrics and Gynecology, Virgen de la Arrixaca, 30120 Murcia, Spain; 9 Units of Reproductive Endocrinology and Human Reproduction, 1st Department of Obstetrics and Gynecology, Aristotle University of Thessaloniki, 54124 Thessaloniki, Greece

**Keywords:** assisted reproductive technology, in vitro fertilization, placenta previa, low-lying placenta, bilobate placenta, single umbilical artery, velamentous cord insertion, vasa previa

## Abstract

**Objective:** Global utilization of assisted reproductive technology (ART) is increasing; however, it is associated with adverse perinatal outcomes. Placental and umbilical cord abnormalities contribute significantly to these negative outcomes. However, it remains unclear whether ART independently increases the risk of such abnormalities. This study aimed to investigate the association between ART and key umbilico-placental abnormalities, after adjustment for confounders. **Methods:** In this retrospective cohort study, singleton pregnancies receiving routine antenatal care (January 2015 to June 2024) at the 3rd Department of Obstetrics and Gynecology, Aristotle University of Thessaloniki, Greece, were analyzed. Pregnancies conceived via ART were compared to those conceived spontaneously. To investigate placental and cord anomalies, this study employed multiple logistic regression. This approach adjusted for various confounders, including maternal age, BMI, parity, smoking status, history of previous cesarean section, diabetes mellitus, and thyroid disease. **Results:** This study included a total of 13,854 singleton pregnancies, of which 647 were conceived via ART. ART was significantly associated with an increased risk of placenta previa (aOR 1.99, 95% CI 1.10–3.61), low-lying placenta (aOR 1.71, 95% CI 1.38–2.11), bilobate placenta (aOR 2.81, 95% CI 1.92–4.11), single umbilical artery (aOR 2.62, 95% CI 1.022–6.715), marginal (aOR 1.63, 95% CI 1.32–2.01) and velamentous cord insertion (aOR 3.13, 95% CI 1.98–4.95), and vasa previa (aOR 5.51, 95% CI 1.28–23.76). **Conclusions:** ART-conceived pregnancies appear to carry a higher risk for certain placental and umbilical cord abnormalities, potentially contributing to adverse perinatal outcomes. Further studies are required to investigate the pathophysiology underlying these associations.

## 1. Introduction

Over the past four decades, assisted reproductive technology (ART) has evolved from an experimental procedure to a mainstream fertility treatment, with more than 10 million babies born via in vitro fertilization (IVF) worldwide [1,2]. This progress has been driven by major technological advances such as intracytoplasmic sperm injection (ICSI), introduced in 1992 to overcome severe male-factor infertility [3]; preimplantation genetic testing, which uses next-generation sequencing to screen embryos for aneuploidies and improve selection [4]; and time-lapse imaging incubators that enable continuous embryo monitoring without disrupting culture conditions, enhancing embryo viability assessment [5]. These advancements have refined ART and improved success rates, leading to increasing global utilization of ART and offering greater hope to individuals and couples facing infertility.

Nevertheless, ART has been associated with various adverse perinatal outcomes in singleton pregnancies. Compared to naturally conceived pregnancies, those conceived via ART demonstrate higher rates of preterm birth, low birth weight, and small-for-gestational-age (SGA) neonates [6], as well as increased maternal complications, such as gestational hypertension, cesarean delivery, and placental disorders [7,8]. However, the exact reasons for these associations remain unclear. It is uncertain how much of the observed risk stems directly from ART-related procedures or whether confounding factors such as advancing maternal age, obesity, and underlying infertility play a predominant role [9]. Additionally, indirect contributors—such as alterations in placental development and umbilical cord abnormalities—may play an unrecognized yet significant role in mediating these adverse outcomes.

Placental and umbilical cord abnormalities are well-established contributors to adverse perinatal outcomes [10,11,12,13]. ART pregnancies may be linked to velamentous and marginal cord insertion both of which are associated with SGA neonates, preterm delivery, preeclampsia, and stillbirth [14,15,16,17]. Other placental abnormalities suspected to be more prevalent among ART pregnancies include low-lying placenta, placenta previa, and placenta accreta—all serious conditions that can compromise placental perfusion and fetal circulation, potentially leading to fetal growth restriction, preterm birth, antepartum hemorrhage, and perinatal mortality [18,19]. Additionally, ART may also be associated with umbilical cord abnormalities such as vasa previa and single umbilical artery [20,21]. Vasa previa is a life-threatening condition that significantly increases the risk of stillbirth and perinatal mortality, while single umbilical artery has been linked to an increased likelihood of SGA neonates [21].

Despite these concerning associations, research on the association of ART with placental and umbilical cord abnormalities remains limited. Few large cohort studies have examined these associations, while most existing studies do not adequately account for key confounders such as maternal age, parity, and BMI. As a result, the true impact of ART on placental and umbilical cord abnormalities remains uncertain.

Therefore, this study aimed to assess the association between ART and several clinically significant placental and umbilical cord abnormalities while adjusting for key confounders. By doing so, we aimed to provide more reliable effect estimates and a clearer understanding of these potential associations.

## 2. Materials and Methods

### 2.1. Study Design and Setting

This retrospective cohort study was conducted from January 2015 to June 2024 at the 3rd Department of Obstetrics and Gynecology, School of Medicine, Faculty of Health Sciences, Aristotle University of Thessaloniki, Greece. Eligible participants were women who conceived through natural conception or ART, as defined by the International Committee for Monitoring Assisted Reproductive Technology, and who received routine antenatal care at the study site.

### 2.2. Ethical and Reporting Standards

The study design and reporting adhered to the Strengthening the Reporting of Observational Studies in Epidemiology (STROBE) guidelines [22], and the analytical approach followed the Transparent Reporting of a Multivariable Prediction Model for Individual Prognosis or Diagnosis (TRIPOD) guidelines [23]. Ethical approval was obtained from the institutional review board of Aristotle University of Thessaloniki (320/2024) on 20 September 2024, and all participants provided informed consent for the use of their anonymized data. For cases where a signed consent form was not available before the retrospective analysis, we contacted the patients directly, invited them for an in-person consultation to provide full information about the study, and obtained written informed consent. This study was conducted in accordance with the Declaration of Helsinki.

### 2.3. Population and Data Collection

In order to create a more homogeneous study population and minimize confounding variables related to different fertility treatments, only singleton pregnancies conceived via IVF or ICSI were included. Pregnancies resulting from ovulation induction or intrauterine insemination were excluded. Participants were required to have a live fetus between 20^+0^ and 23^+6^ weeks of gestation, with no known genetic anomalies or major fetal structural defects. Multiple pregnancies, pregnancy terminations, miscarriages, and cases lost to follow-up were also excluded.

As part of a standardized protocol, maternal demographic, clinical, and lifestyle information (height, weight, age, smoking history, gravidity/parity, and mode of conception) are systematically collected during the initial consultation and entered into the Astraya database. Ultrasound assessments include estimated fetal weight, amniotic fluid index, uterine artery Doppler pulsatility indices, and evaluations of placental and umbilical abnormalities (e.g., placenta previa, bilobate placenta, cord insertion site—central/eccentric, marginal or velamentous, single umbilical artery, and vasa previa). All sonographers are certified by the Fetal Medicine Foundation (London, UK), ensuring standardized scan quality and measurement consistency.

### 2.4. Statistical Analysis

Statistical analyses were performed initially using descriptive tests. Continuous variables were assessed for normality and presented as mean with its 95% confidence intervals (95% CI) if normally distributed, otherwise as median with its 95% CI. Differences between the control and study groups were compared using *t*-tests or one-way Analysis of Variance (ANOVA) for normal variables and the Mann–Whitney U or Kruskal-Wallis test for non-normal variables. Categorical variables, expressed as a percentage with its 95% CI, were compared using the chi-squared test. Pregnancies conceived spontaneously served as the control group, whereas those conceived via ART comprised the study group. Multiple logistic regression was then applied to estimate adjusted odds ratios (aOR) with 95% CI for the association between ART and each placental or umbilical abnormality, adjusting for confounders such as maternal age, parity, smoking status, body mass index (BMI), previous cesarean section, pre-existing diabetes mellitus, and thyroid disease.

Propensity score matching (PSM) was performed as a sensitivity analysis to address any baseline differences between pregnancies conceived spontaneously and those conceived via ART. Propensity scores were computed via multiple logistic regression, incorporating the same set of confounders mentioned above, and matching was conducted in a 1:2 ratio (ART to spontaneous conception). After ensuring adequate balance based on standardized mean differences, conditional logistic regression was performed in the matched sample to re-examine the significant associations found in the original multiple logistic regressions.

All analyses were performed using R version 2.15.1 (R Foundation for Statistical Computing, Vienna, Austria). Statistical significance was set at *p* < 0.05.

## 3. Results

**Figure 1:**
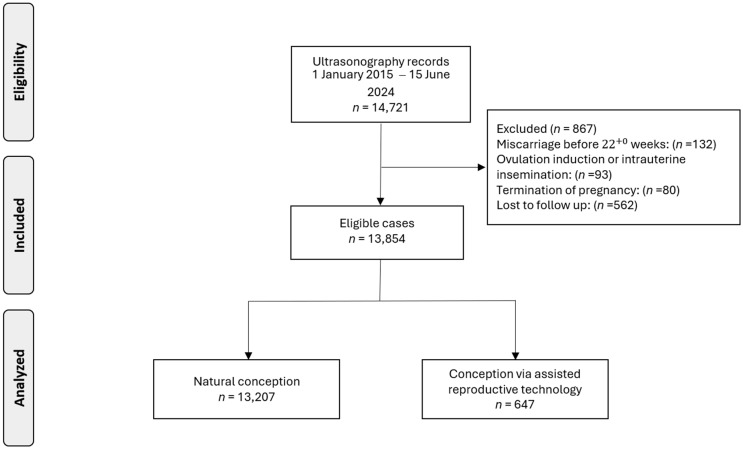
Flowchart of patient inclusion in this study.

Data were collected from 14,721 pregnant women. Following the exclusion of cases due to pregnancy termination (*n* = 80), miscarriage before 22^+0^ weeks (*n* = 132), ovulation induction or intrauterine insemination (*n* = 93), or loss to follow-up (n = 562), our analysis was refined to 13,854 singleton pregnancies, including 647 (4.7%) pregnancies conceived via ART (Figure 1).

Compared to the control group, women who conceived via ART were older and more likely to be nulliparous, non-smokers, and to have diabetes mellitus or thyroid disease (Table 1). Missing data on important variables were noted for less than 4% of the participants. We considered the missing data to be missing at random, as it predominantly occurred in variables of limited significance, such as smoking status. Consequently, these cases were excluded from our analysis.

### 3.1. Multivariable Regression Analyses

In our multivariable analyses, ART was significantly associated with several placental and umbilical abnormalities (Table 2). Detailed multivariable models are provided in Supplementary Tables S1–S11. Women who conceived via ART had approximately three-fold higher odds of bilobate placenta (aOR 2.81, 95% CI 1.92–4.11, *p* < 0.001). Placental attachment near the cervical os was also more common in ART pregnancies. Specifically, placenta previa had nearly double odds (aOR 1.99, 95% CI 1.10–3.61, *p* = 0.023), and low-lying placenta (less than 2 cm than os but not placenta previa) was 70% more likely in ART pregnancies (aOR 1.71, 95% CI 1.38–2.11, *p* < 0.001).

Regarding umbilical cord abnormalities, a single umbilical artery had approximately three-fold higher odds in the ART group (aOR 2.62, 95% CI 1.02–6.72, *p* = 0.045) than in the spontaneous conception group. Marginal cord insertion (aOR 1.63, 95% CI 1.32–2.01, *p* < 0.001) was also more frequent in ART pregnancies. Velamentous cord insertion exhibited an even higher risk, with ART conferring more than triple the odds (aOR 3.13, 95% CI 1.98–4.95, *p* < 0.001). Furthermore, vasa previa was more than five times more common in ART pregnancies (aOR 5.51, 95% CI 1.28–23.76, *p* = 0.022).

### 3.2. Sensitivity Analyses via Propensity Score Matching

**Figure 2:**
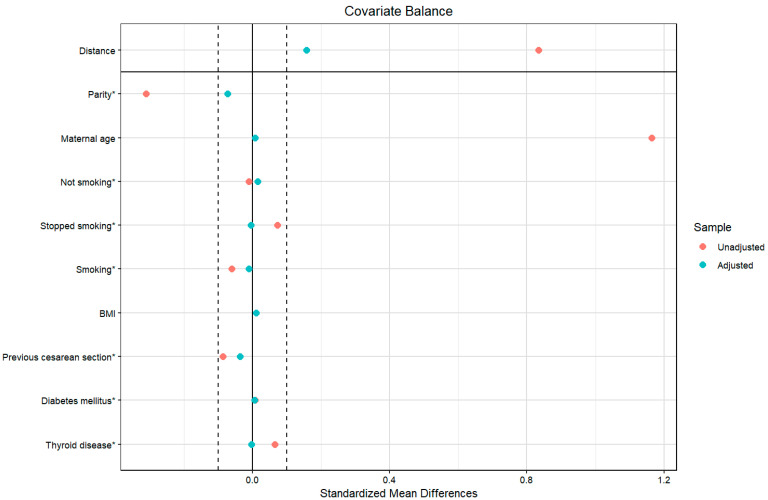
Covariate balance of the investigated populations before and after propensity score matching. Legend: The vertical dashed lines at ±0.1 standardized mean difference represent the conventional threshold for acceptable balance; * denotes the categorical variables.

As a sensitivity analysis for the statistically significant results, we adjusted further for confounders using propensity score matching in all the outcomes; the balancing was successful, and the standardized mean difference of all the confounding variables was less than 0.1 (Figure 2).

After propensity score matching, women who conceived via ART had more than three times higher odds of developing a bilobate placenta than those who conceived spontaneously (aOR 2.85, 95% CI 1.72–4.73, *p* = 0.001). Moreover, women who conceived via ART had approximately two times higher odds of developing low-lying placenta (aOR 1.77, 95% CI 1.36–2.29, *p* = 0.001) and placenta previa (aOR 1.89, 95% CI, 1.03–3.47; *p* = 0.041). Conception via ART doubled the odds of having marginal (aOR 1.76, 95% CI 1.34–2.31, *p* = 0.001) or velamentous cord insertion (aOR 2.08, 95% CI 1.13–3.83, *p* = 0.014). Of note, the mode of conception was not associated with the occurrence of a single umbilical artery (aOR 1.63, 95% CI 0.54–4.86, *p* = 0.384) or vasa previa (aOR 3.61, 95% CI 0.65–19.91, *p* = 0.126) after propensity matching.

## 4. Discussion

### 4.1. Primary Findings

This retrospective cohort study revealed several important findings that link ART to both placental and umbilical cord abnormalities. Specifically, ART conception was significantly associated with higher rates of (i) low-lying placenta, (ii) placenta previa, (iii) bilobate placenta, (iv) single umbilical artery, (v) vasa previa, (vi) marginal, and (vii) velamentous cord insertion.

### 4.2. Interpretation of the Findings

ART pregnancies exhibit notably higher odds of low-lying placenta and placenta previa. A study that investigated the incidence of placenta previa found that compared to spontaneous conception, ART pregnancies had an almost three times higher risk of placenta previa (aOR 2.9, 95% CI 1.4–6.1), which is in accordance with our findings [22]. Additionally, a recent meta-analysis reported a statistically significant association between ART and placenta previa, with an aOR of 3.65; they included 14 studies, each including different confounding factors, resulting in very high heterogeneity [23]. One proposed mechanism is that ART disrupts normal trophoblastic invasion or implantation, favoring placental attachment to the lower uterine segment. In IVF, embryos are placed transcervically with a catheter, which may trigger uterine contractions (possibly through prostaglandin release) and increase lower-segment implantation [24]. Since approximately 80% of embryos implant near the transfer site [25], and lower embryo placement is often preferred to improve intrauterine implantation rates [26], the risk of placenta previa may increase. Other theories point to supraphysiological steroid levels during controlled ovarian stimulation affecting placentation [27]. A thin endometrial lining is also associated with a higher risk of placenta previa in ART pregnancies [28], and controlled ovulation induction may impair endometrial receptivity and trophoblast invasion, with a dose-response effect for hormonal therapy [29,30]. Endometriosis appears to further elevate risk, as shown in a meta-analysis by Jeon et al. [31], and it warrants special attention in ART pregnancies with endometriosis or tubal disease [32]. Dysregulation of HOXA1 and certain endometrial miRNAs may underlie these receptivity issues [30]. Consequently, ART itself may be an independent risk factor for placenta previa, likely because of altered endometrial receptivity and trophoblast erosion.

We further observed a three-fold increase in the risk of bilobate placenta among ART pregnancies, which was confirmed even after PSM. This condition may reflect altered early placental development, possibly due to embryo manipulation or other ART laboratory factors that affect trophoblast differentiation and vascularization. Although this has been under-investigated, two studies have reported a significant positive association between ART and bilobate placenta [33,34]. The proposed mechanism involves the use of assisted hatching, which might accelerate endometrial penetration and disrupt normal placental and umbilical cord formation [35]. Inadequate hatching may also impede complete embryo development, potentially causing placental malformation, miscarriage, or multiple gestations [35]. Because assisted hatching targets the trophectoderm—the future placenta—it may affect these cells and contribute to higher rates of bilobate placenta [36].

In our primary analysis, a single umbilical artery was nearly three times more frequent in ART pregnancies. Although statistical significance was lost following PSM, the elevated effect estimate persisted, suggesting that the loss of significance may be due to the further reduction of an already limited sample size rather than the absence of a true association. While no prior studies have controlled for confounders in this context, they have reported a significant positive association between ART and a single umbilical artery [21,37]. Potential mechanisms linking ART to a single umbilical artery are not well understood but may involve disruptions in early vascular development. Physical manipulation of embryos (e.g., assisted hatching) can alter trophoblastic or mesodermal tissues that eventually form the umbilical cord [38].

Conception via ART was strongly linked to both marginal and velamentous cord insertions, in line with two recent meta-analyses [16,17]. These associations remained after further adjustment for confounders through PSM, highlighting a possible causal association. ART procedures may influence these outcomes by affecting early placental development through the trophotropism and polarity hypotheses [39]. The trophotropism hypothesis proposes that ART-related embryo manipulation causes the placenta to migrate to more vascularized regions during early gestation, enhancing its blood supply but leaving the umbilical cord behind [40]. According to the polarity hypothesis, blastocyst malrotation during ART-assisted implantation forces the umbilical vessels to spread abnormally, leading to atypical cord insertions [39]. Velamentous cord insertion, in particular, increases the risk of vasa previa and fetal vessel rupture, underscoring the importance of early detection.

After adjusting for confounders, we noted that vasa previa was nearly five times more common among ART pregnancies (aOR 5.51). The aOR remained high in the PSM sensitivity analysis when we eliminated the differences between the confounders, although it lost significance due to the limited number of cases. The very low incidence of vasa previa causes wide confidence intervals and limits the robustness of our results. A recent meta-analysis identified a significant association between ART and vasa previa, highlighting low-lying placenta, velamentous cord insertion, and bilobate placenta as the main risk factors [41]. Our analysis also linked ART to these factors, supporting the increased risk of vasa previa in ART pregnancies. According to a study by Attilakos et al., 50% of vasa previa cases are complicated by either a low-lying placenta or velamentous cord insertion [42]. As these risk factors are also more common among ART pregnancies, ART may increase the risk of vasa previa both directly and indirectly. Therefore, in ART pregnancies with these factors, careful scanning for vasa previa may be considered.

All of these associations of ART with clinically significant umbilico-placental abnormalities could partly explain the elevated risk of adverse perinatal outcomes in these pregnancies. Rather than ART itself acting as a direct cause, these placental and umbilical abnormalities may serve as indirect mechanisms leading to complications [43,44]. Abnormal placentation has been suspected as a possible contributor to ART-related issues [11,12,13], and because the placenta is of paramount importance to fetal development, even minor disruptions may significantly affect pregnancy outcomes and potentially have long-term effects on offspring health [45]. Consequently, understanding how ART influences placental structure and function is crucial for assessing its role in perinatal outcomes and for managing these pregnancies more effectively.

### 4.3. Possible Clinical Implications

These findings have several potential clinical implications. First, increased surveillance for placental and cord insertion abnormalities in IVF pregnancies may be warranted. Detailed ultrasound assessments, including transvaginal scans for placenta previa and targeted imaging of the cord insertion site, could facilitate earlier detection and improved management of high-risk cases. Second, awareness of the heightened risk of velamentous and marginal cord insertions could prompt more vigilant prenatal follow-up, thereby potentially reducing adverse outcomes related to fetal growth restriction or vasa previa. Finally, counseling of patients undergoing IVF should reflect these findings, offering individualized risk stratification and emphasizing the importance of comprehensive obstetric care.

### 4.4. Strengths and Limitations

A key strength of this study is the large sample size and systematic approach to data collection. All sonographers were certified by the Fetal Medicine Foundation, ensuring high-quality and standardized ultrasound measurements. Additionally, our use of robust statistical methods—such as multiple logistic regression and propensity score matching—mitigates the impact of known confounders, including maternal age, parity, smoking status, and pre-existing medical conditions.

Nevertheless, several limitations warrant consideration. First, although we accounted for important covariates, residual confounding cannot be entirely ruled out, especially with respect to infertility etiology, specific ART protocols (e.g., fresh vs. frozen embryo transfers, assisted hatching, IVF, and ICSI), and lifestyle factors such as diet and physical activity. Future investigations should categorize ART pregnancies according to these variations to ascertain whether certain techniques exhibit higher risks of placental and umbilical abnormalities. Second, this study focuses on immediate pregnancy outcomes without assessing long-term health implications for offspring; longitudinal research is necessary to clarify the broader consequences of these abnormalities. Third, our single-center design may limit the generalizability of findings to different populations or healthcare contexts, particularly where socioeconomic status and access to healthcare could influence both the likelihood of undergoing IVF and the quality of prenatal care. Fourth, the retrospective nature of this study introduces the potential for missing or incomplete data. Fifth, the use of multiple statistical comparisons increases the likelihood of a Type I error, with an estimated false-positive risk of approximately 40%. Lastly, although we speculate on possible biological pathways linking ART to placental and umbilical abnormalities, we lack direct mechanistic evidence. Future studies employing molecular or genetic analyses could elucidate these underlying mechanisms more definitively.

## 5. Conclusions

In conclusion, the findings of the present study suggest that pregnancies achieved via ART face a higher risk of certain placental and umbilical cord abnormalities, including low-lying placenta, placenta previa, bilobate placenta, marginal or velamentous cord insertions, vasa previa, and single umbilical artery. These findings underscore the need for increased surveillance and targeted ultrasound evaluation in ART-conceived pregnancies. Moreover, counseling patients undergoing ART should reflect these findings, offer individualized risk stratification, and emphasize the importance of comprehensive obstetric care. Future prospective studies with larger populations and more granular ART data could further elucidate the underlying mechanisms and refine the risk stratification strategies for these patients.

## Figures and Tables

**Table 1:** Characteristics of the overall, control, and study populations.

Variable	Level	Overall	Natural Conception	Conception via ART	*p*-Value
		*n* = 13,854	*n* = 13,207	*n* = 647	
Parity ^‡^	Multiparity	47.8% (46.9–48.6%)	49.2% (48.4–50.1%)	18.2% (15.3–21.4%)	<0.001
	NA	0.06% (0.02–0.11%)	0.06% (0.03–0.12%)	0.00% (0.00–0.57%)	
Preexisting Diabetes Mellitus ^‡^		0.39% (0.29–0.51%)	0.36% (0.26–0.47%)	1.08% (0.44–2.22%)	0.012
Thyroid disease ^‡^		8.08% (7.64–8.55%)	7.77% (7.32–8.24%)	14.5% (11.9–17.5%)	<0.001
Maternal Age *		31.4 (31.3–31.5)	31.1 (31.0–31.2)	37.3 (36.9–37.7)	<0.001
BMI ^†^		23.1 (23.0–23.1)	23.1 (23.0–23.1)	23.0 (22.7–23.4)	0.694
Smoking ^‡^	No smoking	57.1% (56.3–58.0%)	57.1% (56.2–57.9%)	58.0% (54.0–61.8%)	<0.001
	Quitted in pregnancy	26.8% (26.1–27.6%)	26.4% (25.7–27.2%)	34.6% (31.0–38.4%)	
	Smoking	12.3% (11.8–12.9%)	12.6% (12.0–13.1%)	6.80% (4.98–9.02%)	
	NA	3.75% (3.44–4.08%)	3.90% (3.58–4.24%)	0.62% (0.17–1.58%)	
Gestational Diabetes Mellitus ^‡^		8.96% (8.49–9.45%)	8.60% (8.13–9.09%)	16.4% (13.6–19.5%)	<0.001
Previous cesarean delivery ^‡^		19.5% (18.8–20.2%)	19.8% (19.2–20.5%)	12.1% (9.65–14.8%)	<0.001
Placental site ^‡^	Anterior	44.8% (43.9–45.6%)	44.9% (44.0–45.8%)	42.2% (38.4–46.1%)	0.004
	Posterior	43.3% (42.5–44.1%)	43.3% (42.5–44.2%)	42.5% (38.7–46.4%)	
	Lateral	9.10% (8.63–9.59%)	8.99% (8.51–9.49%)	11.4% (9.09–14.1%)	
	Fundal	2.47% (2.22–2.74%)	2.46% (2.20–2.74%)	2.63% (1.54–4.17%)	
	Complete Previa	0.38% (0.28–0.49%)	0.33% (0.24–0.45%)	1.24% (0.54–2.42%)	
Lateral Placenta ^‡^		9.10% (8.63–9.59%)	8.99% (8.51–9.49%)	11.4% (9.09–14.1%)	0.040
Placental height from cervical os ^‡^	High	86.0% (85.4–86.6%)	86.6% (86.0–87.2%)	73.9% (70.3–77.2%)	<0.001
	Low-lying	13.1% (12.5–13.6%)	12.6% (12.0–13.1%)	23.3% (20.1–26.8%)	
	Previa	0.93% (0.78–1.11%)	0.84% (0.69–1.01%)	2.78% (1.66–4.36%)	
Bilobate placenta ^‡^		2.16% (1.92–2.41%)	1.94% (1.71–2.19%)	6.65% (4.85–8.85%)	<0.001
Single umbilical artery ^‡^		0.61% (0.49–0.76%)	0.60% (0.47–0.74%)	0.93% (0.34–2.01%)	0.301
Umbilical cord insertion ^‡^	Central/eccentric	86.7% (86.2–87.3%)	87.3% (86.7–87.9%)	75.3% (71.8–78.6%)	<0.001
	Marginal	11.9% (11.4–12.5%)	11.5% (10.9–12.0%)	20.9% (17.8–24.2%)	
	Velamentous	1.34% (1.16–1.55%)	1.22% (1.04–1.42%)	3.86% (2.52–5.65%)	
Vasa previa ^‡^		0.12% (0.07–0.19%)	0.09% (0.05–0.16%)	0.62% (0.17–1.58%)	0.005
UtA PI z ^†^		0.01 (−0.01–+0.02)	0.02 (+0.00–+0.04)	−0.26 (−0.36–−0.17)	<0.001
UtA PI percentile ^†^		47.1 (46.3–48.0)	47.7 (46.8–48.5)	34.1 (29.8–38.4)	<0.001
UtA PI > 95th percentile ^‡^	Above 95th percentile	7.31% (6.88–7.76%)	7.34% (6.90–7.79%)	6.80% (4.98–9.02%)	0.789
	NA	0.08% (0.04–0.14%)	0.08% (0.04–0.15%)	0.00% (0.00–0.57%)	
Uterine Artery Notch ^‡^	None	93.9% (93.5–94.3%)	93.9% (93.4–94.3%)	95.4% (93.4–96.9%)	0.361
	Unilateral	4.52% (4.18–4.88%)	4.55% (4.20–4.92%)	3.86% (2.52–5.65%)	
	Bilateral	1.49% (1.29–1.70%)	1.52% (1.32–1.75%)	0.77% (0.25–1.79%)	
	NA	0.06% (0.02–0.11%)	0.06% (0.03–0.12%)	0.00% (0.00–0.57%)	

Abbreviations: UtA PI, uterine artery pulsatility index; NA, not available. *, normal continuous variable expressed as mean and 95% confidence intervals; ^†^, non-normal continuous variable expressed as median and 95% confidence intervals; ^‡^, categorical variable expressed as percentage and 95% confidence intervals.

**Table 2:** Cumulative results of the multivariable regressions on the association between the mode of conception and the investigated placental and umbilical variations.

Variable	aOR	95% CI	*p*-Value
Bilobate placenta	2.81	1.92–4.11	0.000
Fundal vs. anterior placenta	1.45	0.84–2.52	0.183
Posterior vs. anterior placenta	1.06	0.88–1.27	0.567
Lateral vs. anterior placenta	1.12	0.84–1.49	0.424
Lateral vs. non-lateral placenta	1.08	0.82–1.41	0.587
Placenta previa vs. high placenta	1.99	1.10–3.61	0.023
Low-lying vs. high placenta	1.71	1.38–2.11	<0.001
Single umbilical artery	2.62	1.02–6.72	0.045
Marginal vs. Central/eccentric cord insertion	1.63	1.32–2.01	<0.001
Velamentous vs. Central/eccentric cord insertion	3.13	1.98–4.95	<0.001
Vasa previa	5.51	1.28–23.76	0.022

Abbreviations: aOR, adjusted odds ratio; CI, confidence intervals; PI, pulsatility index. Multivariable logistic regressions adjusting for maternal age, BMI, multiparity, smoking, history of previous cesarean section, diabetes mellitus, and thyroid disease.

## Data Availability

The data presented in this study are available on request from the corresponding author. The data are not publicly available due to privacy restrictions.

## Supplementary Materials

The following supporting information can be downloaded at: https://www.mdpi.com/article/10.3390/jpm15050176/s1, Supplementary Table S1. Multivariable logistic regression investigating the association between ART and bilobate placenta; Supplementary Table S2. Multivariable logistic regression investigating the association between ART and fundal placental position using anterior position as reference; Supplementary Table S3. Multivariable logistic regression investigating the association between ART and posterior placental position using anterior position as reference; Supplementary Table S4. Multivariable logistic regression investigating the association between ART and lateral placental position using anterior position as reference; Supplementary Table S5. Multivariable logistic regression investigating the association between ART and lateral placental position using all other positions as reference; Supplementary Table S6. Multivariable logistic regression investigating the association between ART and placenta previa using placentas with bigger distance than 2 cm from the cervix os as reference; Supplementary Table S7. Multivariable logistic regression investigating the association between ART and low-lying placenta using placentas with bigger distance than 2 cm from the cervix os as reference; Supplementary Table S8. Multivariable logistic regression investigating the association between ART and single umbilical artery; Supplementary Table S9. Multivariable logistic regression investigating the association between ART and marginal cord insertion using central/eccentric cord insertion as reference; Supplementary Table S10. Multivariable logistic regression investigating the association between ART and velamentous cord insertion using central/eccentric cord insertion as reference; Supplementary Table S11. Multivariable logistic regression investigating the association between ART and vasa previa.

## Abbreviations

The following abbreviations are used in this manuscript:

ART	Assisted reproductive technology
IVF	In vitro fertilization
ICSI	Intracytoplasmic sperm injection
SGA	Small for gestational age
PSM	Propensity score matching
