# Supplementary material for: The Association of Assisted Reproductive Technology with Placental and Umbilical Abnormalities
Source: J Pers Med. 2025 Apr 27;15(5):176. doi: 10.3390/jpm15050176 (PMC12113098; doi:10.3390/jpm15050176)
Supplement: Supplementary file 1 [file jpm-15-00176-s001.zip › jpm-3599172-supplementary.pdf]

Journal of Personalized Medicine

Article

The Association of Assisted Reproductive Technology with Placental and Umbilical Abnormalities

Antonios Siargkas [1], Ioannis Tsakiridis [1], Sonia Giouleka [1], Petya Chaveeva [2,3], Maria Mar Gil [4,5], Walter Plasencia [6], Catalina De Paco Matallana [7,8], Efstratios M. Kolibianakis [9,†] and Themistoklis Dagklis [1,*,†]

Table S1. Multivariable logistic regression investigating the association between ART and bilobate placenta.

Variable	aOR	95% CI	*p*–value
ART	2.80	1.92–4.11	0.000
Parity	0.90	0.67–1.20	0.475
Maternal age	1.03	1.01–1.06	0.008
Ref: No smoking			
Stopped Smoking	0.94	0.72–1.23	0.649
Smoking	1.06	0.75–1.51	0.743
BMI	1.00	0.98–1.02	0.973
History of previous CS	1.13	0.81–1.59	0.473
Diabetes mellitus	1.40	0.33–5.88	0.644
Thyroid disease	0.70	0.44–1.10	0.117

Abbreviations: aOR, adjusted odds ratio; ART, assisted reproductive technology; CI, confidence intervals; CS, cesarean section.

Table S2. Multivariable logistic regression investigating the association between ART and fundal placental position using anterior position as reference.

Variable	aOR	95% CI	*p*–value
ART	1.45	0.84–2.52	0.183
Parity	1.20	0.90–1.61	0.212
Maternal age	0.98	0.96–1.01	0.118
Ref: No smoking			
Stopped Smoking	1.01	0.77–1.33	0.925
Smoking	1.03	0.72–1.47	0.889
BMI	1.03	1.01–1.05	0.006
History of previous CS	1.01	0.72–1.40	0.972
Diabetes mellitus	2.13	0.48–9.36	0.319
Thyroid disease	0.86	0.55–1.34	0.501

Abbreviations: aOR, adjusted odds ratio; ART, assisted reproductive technology; CI, confidence intervals; CS, cesarean section.

 https://doi.org/10.3390/jpm15050176

Table S3. Multivariable logistic regression investigating the association between ART and posterior placental position using anterior position as reference.

Variable	aOR	95% CI	*p*–value
ART	1.06	0.88–1.27	0.567
Parity	1.05	0.96–1.15	0.258
Maternal age	1.00	0.99–1.01	0.523
Ref: No smoking			
Stopped Smoking	1.03	0.95–1.12	0.507
Smoking	1.08	0.97–1.21	0.175
BMI	1.00	0.99–1.01	0.804
History of previous CS	0.94	0.84–1.05	0.243
Diabetes mellitus	1.66	0.90–3.05	0.103
Thyroid disease	0.98	0.86–1.12	0.802

Abbreviations: aOR, adjusted odds ratio; ART, assisted reproductive technology; CI, confidence intervals; CS, cesarean section.

Table S4. Multivariable logistic regression investigating the association between ART and lateral placental position using anterior position as reference.

Variable	aOR	95% CI	*p*–value
ART	1.12	0.85–1.49	0.424
Parity	0.86	0.74–1.00	0.050
Maternal age	1.01	0.99–1.02	0.213
Ref: No smoking			
Stopped Smoking	1.07	0.93–1.23	0.343
Smoking	0.82	0.67–1.01	0.067
BMI	1.00	0.99–1.02	0.612
History of previous CS	0.91	0.75–1.10	0.334
Diabetes mellitus	1.81	0.74–4.40	0.191
Thyroid disease	1.32	1.08–1.62	0.007

Abbreviations: aOR, adjusted odds ratio; ART, assisted reproductive technology; CI, confidence intervals; CS, cesarean section.

Table S5. Multivariable logistic regression investigating the association between ART and lateral placental position using all other positions as reference.

Variable	aOR	95% CI	*p*–value
ART	1.08	0.82–1.41	0.587
Parity	0.84	0.72–0.97	0.016
Maternal age	1.01	0.99–1.02	0.261
Ref: No smoking			
Stopped Smoking	1.05	0.92–1.20	0.444
Smoking	0.78	0.64–0.96	0.017
BMI	1.00	0.99–1.01	0.711
History of previous CS	0.94	0.78–1.12	0.469
Diabetes mellitus	1.40	0.63–3.12	0.413
Thyroid disease	1.34	1.11–1.63	0.002

Abbreviations: aOR, adjusted odds ratio; ART, assisted reproductive technology; CI, confidence intervals; CS, cesarean section.

Table S6. Multivariable logistic regression investigating the association between ART and placenta previa using placentas with bigger distance than 2cm from the cervix os as reference.

Variable	aOR	95% CI	*p*–value
ART	1.99	1.10–3.61	0.023
Parity	0.85	0.54–1.35	0.498
Maternal age	1.11	1.07–1.16	0.000
Ref: No smoking			
Stopped Smoking	0.83	0.55–1.27	0.392
Smoking	1.07	0.63–1.83	0.800
BMI	0.99	0.95–1.03	0.560
History of previous CS	1.51	0.92–2.46	0.103
Diabetes mellitus	1.35	0.18–10.15	0.771
Thyroid disease	0.81	0.43–1.52	0.503

Abbreviations: aOR, adjusted odds ratio; ART, assisted reproductive technology; CI, confidence intervals; CS, cesarean section.

Table S7. Multivariable logistic regression investigating the association between ART and low–lying placenta using placentas with bigger distance than 2cm from the cervix os as reference.

Variable	aOR	95% CI	*p*–value
ART	1.71	1.38–2.11	0.000
Parity	0.98	0.86–1.11	0.719
Maternal age	1.04	1.03–1.06	0.000
Ref: No smoking			
Stopped Smoking	1.00	0.89–1.13	0.996
Smoking	1.08	0.92–1.27	0.350
BMI	0.99	0.97–0.99	0.006
History of previous CS	0.84	0.71–0.98	0.023
Diabetes mellitus	0.89	0.37–2.09	0.780
Thyroid disease	0.82	0.68–0.99	0.049

Abbreviations: aOR, adjusted odds ratio; ART, assisted reproductive technology; CI, confidence intervals; CS, cesarean section.

Table S8. Multivariable logistic regression investigating the association between ART and single umbilical artery.

Variable	aOR	95% CI	*p*–value
ART	2.62	1.02–6.72	0.045
Parity	0.96	0.50–1.86	0.913
Maternal age	0.99	0.94–1.04	0.655
Ref: No smoking			
Stopped Smoking	0.92	0.50–1.69	0.784
Smoking	1.11	0.51–2.41	0.794
BMI	0.98	0.93–1.04	0.529
History of previous CS	1.16	0.53–2.5	0.715
Diabetes mellitus	0.00	0.00–Inf	0.982
Thyroid disease	0.38	0.09–1.56	0.178

Abbreviations: aOR, adjusted odds ratio; ART, assisted reproductive technology; CI, confidence intervals; CS, cesarean section.

Table S9. Multivariable logistic regression investigating the association between ART and marginal cord insertion using central/eccentric cord insertion as reference.

Variable	aOR	95% CI	*p*–value
ART	1.63	1.32–2.01	0.000
Parity	0.81	0.71–0.92	0.002
Maternal age	1.01	1.003–1.02	0.027
Ref: No smoking			
Stopped Smoking	1.00	0.88–1.12	0.955
Smoking	0.89	0.75–1.05	0.158
BMI	1.01	1.00–1.02	0.062
History of previous CS	0.95	0.80–1.11	0.494
Diabetes mellitus	1.34	0.65–2.78	0.429
Thyroid disease	1.19	0.99–1.42	0.052

Abbreviations: aOR, adjusted odds ratio; ART, assisted reproductive technology; CI, confidence intervals; CS, cesarean section.

Table S10. Multivariable logistic regression investigating the association between ART and velamentous cord insertion using central/eccentric cord insertion as reference.

Variable	aOR	95% CI	*p*–value
ART	3.13	1.98–4.95	0.000
Parity	0.75	0.52–1.09	0.137
Maternal age	1.02	0.99–1.05	0.225
Ref: No smoking			
Stopped Smoking	1.07	0.77–1.49	0.679
Smoking	0.97	0.60–1.55	0.888
BMI	1.00	0.96–1.02	0.476
History of previous CS	0.90	0.54–1.41	0.581
Diabetes mellitus	3.17	0.74–13.48	0.119
Thyroid disease	0.55	0.29–1.06	0.072

Abbreviations: aOR, adjusted odds ratio; ART, assisted reproductive technology; CI, confidence intervals; CS, cesarean section.

Table S11. Multivariable logistic regression investigating the association between ART and vasa previa.

Variable	aOR	95% CI	*p*–value
ART	5.51	1.28–23.76	0.022
Parity	1.59	0.47–5.37	0.454
Maternal age	1.06	0.95–1.18	0.281
Ref: No smoking			
Stopped Smoking	1.97	0.63–6.20	0.245
Smoking	1.78	0.36–8.92	0.483
BMI	0.99	0.90–1.11	0.957
History of previous CS	0.23	0.03–1.99	0.182
Diabetes mellitus	0.00	0.00–Inf	0.989
Thyroid disease	1.39	0.31–6.34	0.671

Abbreviations: aOR, adjusted odds ratio; ART, assisted reproductive technology; CI, confidence intervals; CS, cesarean section.
